# Characteristics of gut microbiota in captive Asian elephants (*Elephas maximus*) from infant to elderly

**DOI:** 10.1038/s41598-023-50429-1

**Published:** 2023-12-27

**Authors:** Sarisa Klinhom, Sirawit Sriwichaiin, Sasiwan Kerdphoo, Jaruwan Khonmee, Nipon Chattipakorn, Siriporn C. Chattipakorn, Chatchote Thitaram

**Affiliations:** 1 Center of Elephant and Wildlife Health, Animal Hospital, Faculty of Veterinary Medicine, Chiang Mai University, Chiang Mai, 50100 Thailand; 2 Cardiac Electrophysiology Unit, Department of Physiology, Faculty of Medicine, Chiang Mai University, Chiang Mai, 50200 Thailand; 3 Center of Excellence in Cardiac Electrophysiology Research, Chiang Mai University, Chiang Mai, 50200 Thailand; 4 Neurophysiology Unit, Cardiac Electrophysiology Research and Training Center, Faculty of Medicine, Chiang Mai University, Chiang Mai, 50200 Thailand; 5 Department of Veterinary Bioscience and Veterinary Public Health, Faculty of Veterinary Medicine, Chiang Mai University, Chiang Mai, 50100 Thailand; 6 Department of Oral Biology and Diagnostic Sciences, Faculty of Dentistry, Chiang Mai University, Chiang Mai, 50200 Thailand; 7 Faculty of Veterinary Medicine, Chiang Mai University, Chiang Mai, 50100 Thailand; 8 Elephant, Wildlife and Companion Animals Research Group, Chiang Mai University, Chiang Mai, 50100 Thailand

**Keywords:** Microbiology, Molecular biology, Zoology

## Abstract

Gut microbiota play an important role in the health and disease of Asian elephants, however, its characteristics at each stage of life have not been thoroughly investigated in maintaining and regulating health of elephants. This study, therefore, aimed to characterize the profiles of the gut microbiota of captive Asian elephants from infants to the elderly. Gut microbiota were identified by 16S rRNA sequencing from the feces of captive Asian elephants with varying age groups, including infant calves, suckling calves, weaned calves, subadult and adult elephants, and geriatric elephants. The diversity of the gut microbiota was lowest in infants, stable during adulthood, and slightly decreased in the geriatric period. The gut microbiota of the infant elephants was dominated by milk-fermenting taxa including genus *Bifidobacterium* of family *Bifidobacteriaceae* together with genus *Akkermansia*. The fiber-fermenting taxa such as *Lachnospiraceae_NK3A20_group* were found to be increased in suckling elephants in differential abundance analysis by Analysis of Compositions of Microbiomes with Bias Correction (ANCOM-BC). The gut microbiota profiles after weaning until the adult period has been uniform as indicated by no significant differences in beta diversity between groups. However, the composition of the gut microbiota was found to change again in geriatric elephants. Understanding of the composition of the gut microbiota of captive Asian elephants at various life stages could be beneficial for promoting good health throughout their lifespan, as well as ensuring the welfare of captive elephants.

## Introduction

Asian elephants (*Elephas maximus*), originally from the wild, have been captured for use and domesticated under human care for thousands of years. Elephants, as herbivores and hindgut fermenters, efficiently process large quantities of low-quality forage. Despite differences in gastrointestinal anatomy between horses and elephants, horse physiology was commonly used as a digestive model. Nonetheless, for both horses and elephants, the proximal colon appears to play a more vital role in fermentation than the cecum^1^. These herbivores obtain their energy through microbial breakdown, a process largely facilitated by the large community of microorganisms residing in the colon or caecum, widely known as the ‘gut microbiota’^2^.

The gut microbiota contributes to gastrointestinal (GI) physiology by maintaining the structural integrity of the mucosal barrier, protecting against pathogens, and developing immune functions all of which have impact on host health^3,4^. The gut microbiota has been shown to facilitate the fermentation of a hitherto indigestible plant diet to short-chain fatty acids (SCFAs) that provide the body with maximum energy and essential nutrients from the dietary fiber, which are crucial for herbivores^5^. The composition of the gut microbiota is influenced by several factors, including diet and age^6^. The initial colonization of the mammalian gut by the microbes occurs shortly after birth^7,8^. Subsequently, the diversity and composition of gut microbiota in herbivores are subject to change depending on the developmental period and environmental factors. During the growth and development of the animal, each stage of growth requires different types of nutrients/food consumption, for example during progression from breastfeeding to roughage in elephants, resulting in changes in the diversity of gut microbiota in each growth period^9^. However, information about the transition of gut microbiota profiles of elephants in different periods of growth and development is still limited.

The deviation of the composition of gut microbiota from normal conditions or ‘gut dysbiosis’ might impair the normal GI physiology and might greatly influent the health status of elephants which mainly relies on the functioning of the gut microbiota. Gut dysbiosis in animals is associated with GI disorders, including GI stasis, indigestion, constipation, enteritis, colic, and diarrhea^10–12^. Notably, GI disorders are also one of the most common disorders found in captive elephants, resulting in the illness or even death of the elephants^13^. Therefore, a study into the imbalance of gut microbiota in elephants might increase our understanding of these conditions. However, this is difficult as the characteristics of the gut microbiota of healthy elephants as a reference for what purports as normal composition have not been thoroughly investigated.

A few studies about gut microbiota had been performed in captive^14–18^ and wild Asian elephants^19^ and also in African species^20,21^. However, those previous studies were mainly conducted with limited samples in zoos. Interestingly, the majority of captive elephants in Thailand are held in semi-free-ranging conditions, which differ in nutrition, water, and environmental factors from the conditions in a zoo. Therefore, the present study aims to characterize the profiles of gut microbiota of different ages of Asian captive elephants in Northern Thailand by using the next-generation sequencing (NGS) technique. In addition, the associations between gut microbiota profiles in healthy adult elephants with several blood parameters, including hematological parameters, liver and kidney functions, and lipid profile, were also investigated. Understanding the normal profiles of gut microbiota at each stage of life would help to determine the factors associated with health issues in Asian elephants in the future.

## Results

### Alpha diversity and beta diversity were significantly different among age groups

**Figure 1:**
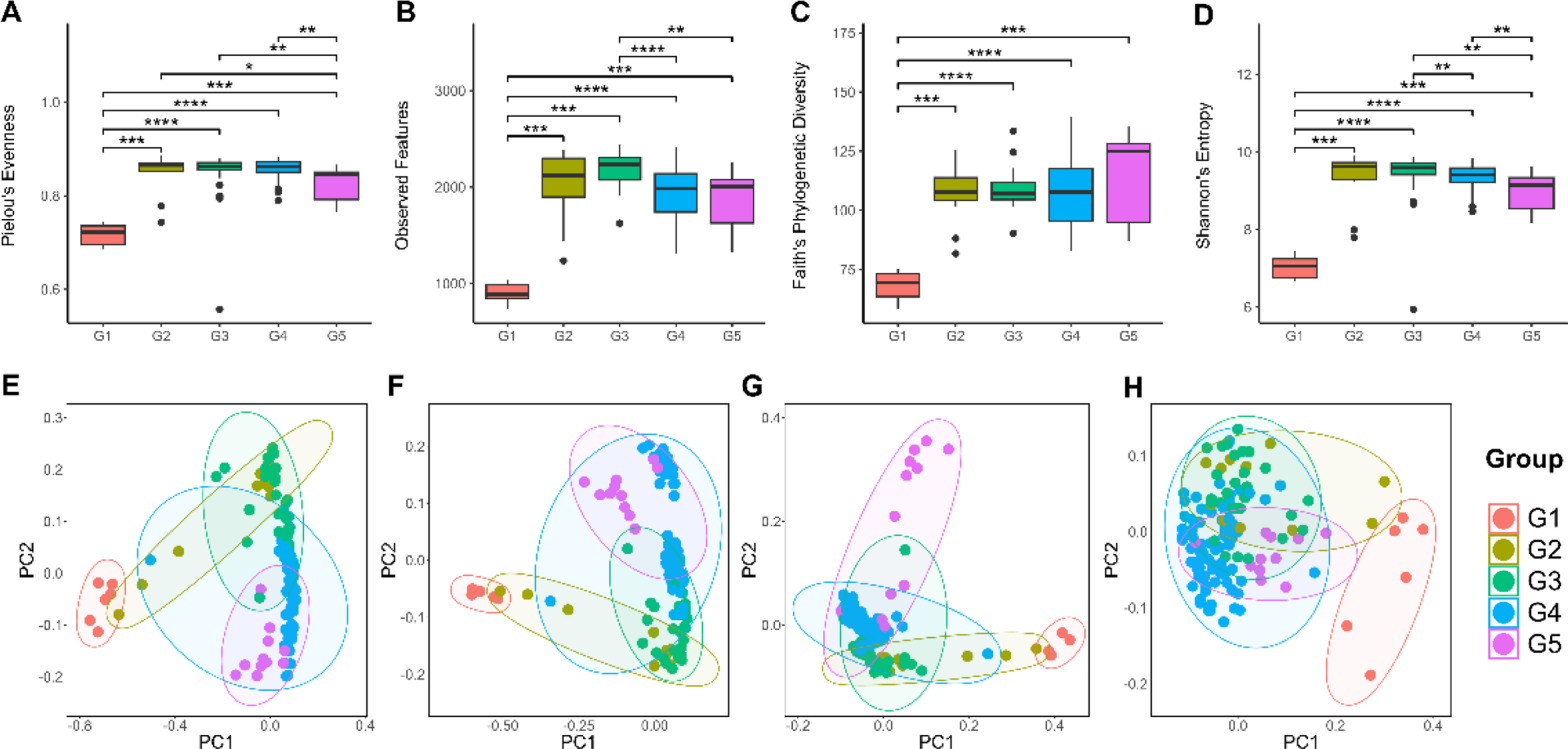
Alpha diversity and beta diversity of gut microbiota of healthy captive elephants in Northern Thailand categorized by age group (Group 1—infant calves; Group 2—suckling calves; Group 3—weaned calves; Group 4—subadult and adult elephants; Group 5—geriatric elephants). (**A–D**) Alpha diversity including (**A**) Pielou’s evenness, (**B**) Observed feature, (**C**) phylogenetic diversity, and (**D**) Shannon’s index. (**E–H**) Beta diversity including (**E**) Bray curtis, (**F**) Jaccard, (**G**) unweighted UniFrac, and (**H**) weighted UniFrac. *p < 0.05, **p < 0.01, ***p < 0.001.

The diversity of gut microbiota in each age group were determined and are presented in terms of alpha diversity in Fig. 1. Infant calves exhibited the lowest fecal microbial alpha diversity when compared to other groups *(*Fig. 1A–D*)*. In addition, geriatric elephants showed a significant decrease in alpha diversity when compared to subadult and adult elephants, as evidenced by Pielou’s evenness *(*Fig. 1A*)* and Shannon’s index *(*Fig. 1D*)*. Notably, the alpha diversity of weaned calves was significantly higher than those of subadult and adult elephants and geriatric elephants as shown in observed feature *(*Fig. 1B*)* and by Shannon’s index *(*Fig. 1D*).*

The similarities of the composition of gut microbiota between groups were calculated based on beta diversity and are presented as principal coordinate analysis (PCoA) plots (Fig. 1). PCoA plots based on Bray–Curtis, Jaccard, unweighted and weighted UniFrac revealed that the gut microbiome of the elephants showed distinct gut microbiota composition across the different age ranges based on p-value of pseudo-F in pairwise PERMANOVA test < 0.05 in pairwise comparison of all groups *(*Fig. 1E–H*)*. In addition, permutational dispersion tests on beta diversity were also conducted by using PERMDISP *(*Supplementary Table 1*)*. The significant of permutational dispersion tests indicated that the distinction of gut microbiota between age group also affected by unequal dispersion between group in addition to real biological differences.

PCoA plots showed the patterns associated with the age group of elephants. The groups of elephants with adjacent ages had a closed distance between the groups in the PCoA plot, suggesting that there was a similar composition of the gut microbiota of the elephants across the adjacent ages. These patterns were observed in PCoA plots following analysis using Bray–Curtis, Jaccard, and unweighted UniFrac distances *(*Fig. 1E–G*)*.

The mantel tests between beta diversity and age were conducted to investigate the overall association of gut microbiota composition with age. Significant correlations between age and beta diversities were found in all measures, indicating that as differences in age become greater there is greater change in the microbiome composition (Supplementary Table 2). The analysis of differential abundance between age groups was conducted by using ANCOM-BC. The results of significantly different taxa of fecal microbiota among age groups with top 10 of log fold changes are presented in Fig. 3. All significantly different taxa are shown in Supplementary Table 3. We used subadult and adult elephants as a reference for each comparison.

### Taxonomic composition of bacterial populations in elephants

**Figure 2:**
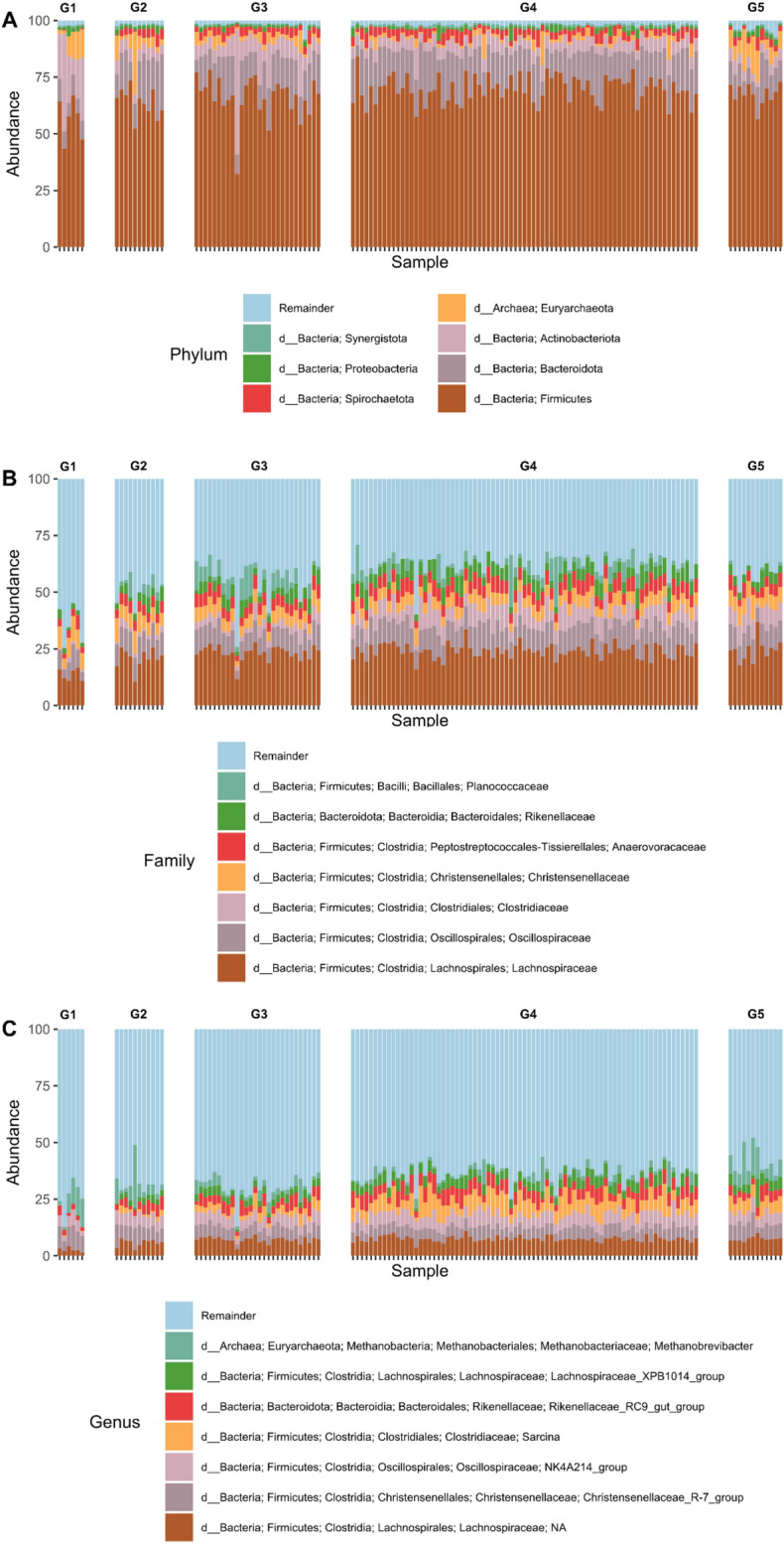
Relative abundance of gut microbiota of captive Asian elephants in Northern Thailand at phylum (**A**), family (**C**), and genus (**B**) level. Elephants categorized by age group (Group 1—infant calves; Group 2—suckling calves; Group 3—weaned calves; Group 4—subadult and adult elephants; Group 5—geriatric elephants).

The taxonomy of gut microbiota was identified based on the data of hypervariable region V3-V4 of the 16 s rRNA gene. Forty-three phyla and 1,134 genera of gut microflora were identified within total elephant fecal samples and details of genera is described in Table 1. The relative abundance at the phylum, family, and genus levels of gut microbiota in all age groups are shown in Fig. 2. The dominant bacterial phyla in fecal samples of all groups of elephants were Firmicutes followed by Bacteroidetes and Actinobacteria. In the infant calves, phylum Bacteroidetes and Spirochete, as well as the families *Oscillospiraceae* and *Clostridiaceae*, along with the genus *Lachnospiraceae; NA*, *Sarcina*, and *Lachnospiraceae_XPB1014_group* of class Clostridia, displayed a low relative abundance. On the other hand, the relative abundance of Actinobacteria and Euryarchaeota were abundantly detected in the infant calves. The composition of the elephant's gut microbiota seemed to be changed from infants to suckling calves, and from subadult and adult to geriatric elephants. The relative abundance of fecal microbiota at the phylum level was shown to be only slightly altered from suckling calves to weaned calves and subadult and adult elephants.

**Table 1:** The abundance of elephant gut microbiota at phylum and genus levels, categorized by age group.

Group	Phylum	Genus
Infant calves	23	409
Suckling calves	26	439
Weaned calves	33	629
Subadult and adult elephant	29	582
Geriatric elephants	41	842

According to the information from human guts, the microbiota is practically stable in healthy adults^22^. This phenomenon was similar to Asian elephants, which reach maturity between 10 and 14 years of age^23^. In this study, the relative abundance of the taxa in the family and genus level of each elephant in the subadult and adult age group are shown in Fig. 2. At the family level of subadult and adult elephants, *Lachnospiraceae* was the most dominant family, followed by *Oscillospiraceae*, *Clostridiaceae*, *Christrensenellacease*, *Anaerovoraceae*, and *Rikenellaceae* (Fig. 2B). The beneficial bacteria were present in fecal samples of subadult and adult elephants, including the fiber-digesting taxa at phylum level of Firmicutes, Bacteroides, Spirochaetota, and Actobacteriota, also including genus *Lachnospiraceae; NA*, *Christrensenellacease_R-7_group*, *NK4A214_group*, *Sarcina*, *Lachnospiraceae_XPB1014_group*. In addition, Archaea *Methanobrevibacter spp.* of phylum Euryarchaeota were observed to be less abundant in subadult and adult elephants (Fig. 2C). In this study, we used subadult and adult elephants as reference for the composition of gut microbiota in healthy elephants for further analyses.

### Infant elephants showed a distinctly different composition of gut microbiota, when compared with subadult and adult elephants

**Figure 3:**
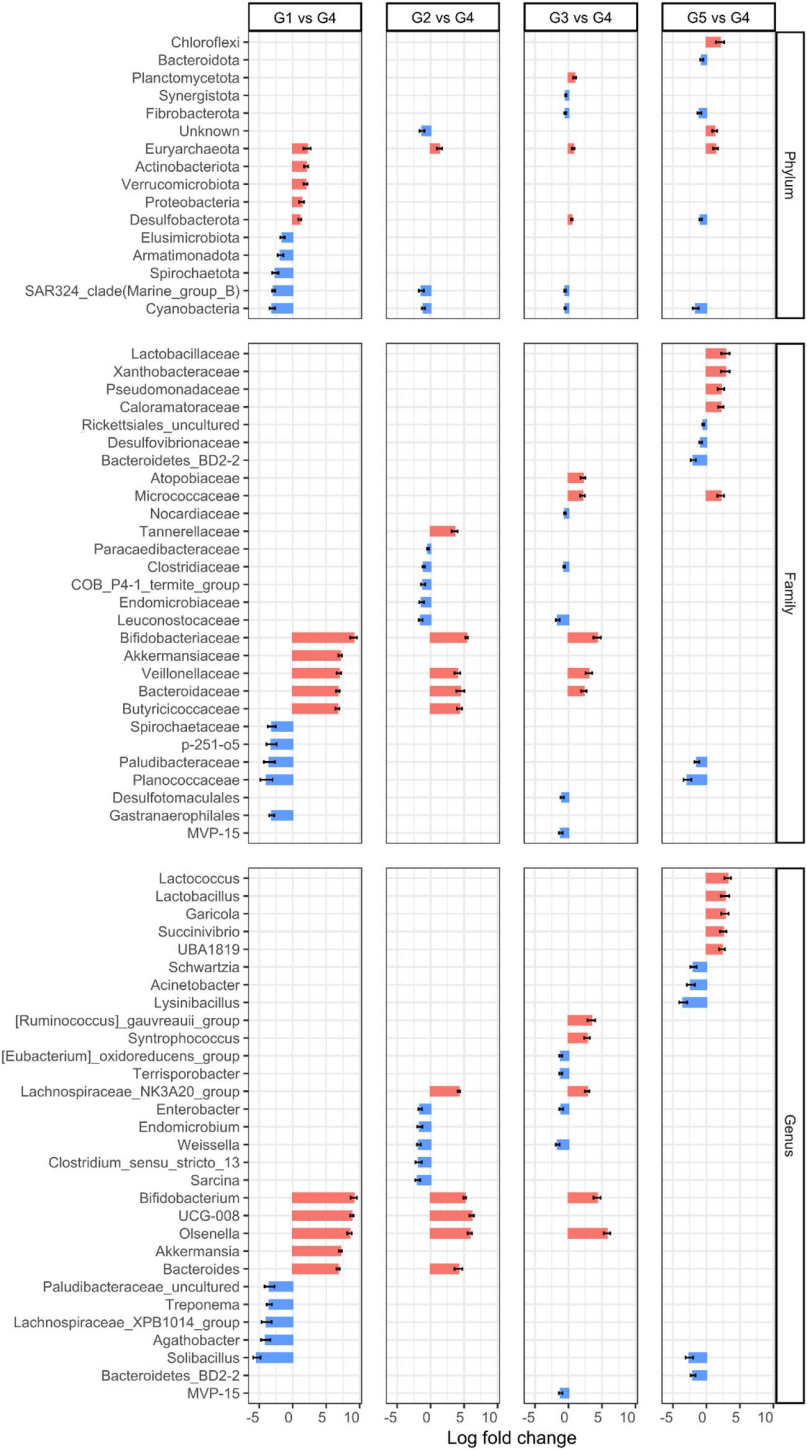
The differential abundance of gut microbiota of healthy captive elephants in Northern Thailand between age groups (Group 1—infant calves; Group 2—suckling calves; Group 3—weaned calves; Group 4—subadult and adult elephants; Group 5—geriatric elephants). The data are presented as Log fold change by using Group 4 as the reference group. Full lists of significant differential abundances are provided in Supplementary Table 3.

The analysis of differential abundance was conducted by using ANCOM-BC. The results of significantly different taxa of fecal microbiota among age groups with top 10 of log fold changes are presented in Fig. 3. All significantly different taxa are shown in Supplementary Table 3. Based on our study's findings, which indicate that the microbiota remains relatively stable in healthy adults, we used subadult and adult elephants as reference points for each comparison.

In the infant calves, the abundance of the phyla Euryarchaeota, Actinobacteriota, Verrucomicrobiota, Proteobacteria, and Desulfobacterota were higher than that of subadult and adult elephants while the phyla Cyanobacteria, SAR324_clade (Marine_group_B), Spirochaetota, Armatimonadota, and Elusimicrobiota were found to be lower (Fig. 3). At the family and genus levels, the taxa from the families *Bifidobacteriaceae, Akkermansiaceae, Villonellaceae, Bacteroidaceae,* and *Butyricioccaceae,* together with the genus *Bifidobacterium, UCG-008, Olsenella, Akkermansia*, and *Bacteroides* in infant calves were higher than those of subadult and adult elephants (Fig. 3). On the other hand, the families *Spirochaetaceae*, *p-251-o5*, *Paludibacteraceae*, *Planococcaceae*, and *Gastranaerophilales*, and the genus *Solibacillus*, *Agathobacter*, *Lachnospiraceae_XPB1014_group*, *Treponema*, and *Paludibacteraceae_ uncultured* were lower in infant calves when compared to subadult and adult elephants (Fig. 3).

### Suckling and weaned elephants showed slightly different gut microbiota composition when compared with adult elephants

When compared to the differences between the infant calves and subadult and adult elephants, both the number of statistically distinct taxa and their magnitude were much smaller between the suckling and weaned calves and subadult and adult elephants. At the phylum level, only Euryarchaeota was higher in suckling calves and Planctomycetota, Euryarchaeota, and Desulfobacterota were higher in weaned calves when compared to subadult and adult elephants (Fig. 3). On the other hand, Synergistota and Fibrobacterota were lower in weaned calves while Cyanobacteria and SAR324_clade (Marine_group_B) were decreased in both suckling and weaned calves (Fig. 3) when compared to subadult and adult elephants.

At the family level, *Bifidobacteriaceae*, *Veillonellaceae*, and *Bacterroidaceae* were higher in both suckling and weaned calves whereas *Tannerellaceae* and *Butyricicoccaceae* were higher only in sucking calves, and *Atopobiaceae* and *Micrococcaceae* were higher only in weaned calves (Fig. 3) when compared to subadult and adult elephants. However, the families *Paracaedibacteriaceae*, *Clostridiaceae*, *COB_P4-1_termite_group*, *Endomicrobiaceae*, and *Leuconostocaceae* in suckling calves, and *Nocardiaceae*, *Clostridiaceae*, *Leuconostocaceae*, *Desulfotomaculales*, and *MVP-15* in weaned calves were lower than in those subadult and adult elephants (Fig. 3).

At the genus level, *Bifidobacterium*, *Olsenella*, and *Lachnospiraceae_NK3A20_group* were higher in sucking and weaned calves while *Bacteroides* and *UCG-008* were higher only in suckling calves, and the *[Ruminococcus]_gauvreauii_group* and *Syntrophococcus* were higher only in weaned calves (Fig. 3). Meanwhile, the genera *Enterobacter*, *Endomicrobium*, *Weissella*, *Clostridium_sensu_stricto_13*, and *Sarcina* in suckling calves, and *[Eubacterium]_oxidoreducens_group*, *Terrisporobacter*, *Enterobacter*, *Weissella*, and *MVP-15* in weaned calves were found to be present lower compared to those in subadult and adult elephants (Fig. 3).

### Geriatric elephants showed distinct gut microbiota, when compared with adult elephants

Geriatric elephants showed a distinct pattern of abundance within the gut microbiota when compared to infant, suckling, and weaned calves. At the phylum level, Chloroflexi, and Euryarchaeota were increased while Cyanobateria, Desulfobacteria, Fibrobacterota, and Bacteroidota were decreased when compared to subadult and adult elephants (Fig. 3).

At the family level, *Lactobacillaceae, Xanthobacteraceae, Pseudomonadaceae, Caloramatoraceae,* and *Micrococcaceae* were more abundant than in subadult and adult elephants. Meanwhile, the taxa from families *Proteobacteria_Rickettsiales_uncultured*, *Desulfovibrionaceae, Bacteroidetes_BD2-2, Paludibacteraceae,* and *Planococcaceae* were lower in composition when compared to those of subadult and adult elephants (Fig. 3).

At the genus level, *Lactococcus*, *Lactobacillus*, *Garicola*, *Succinivibrio*, and *UBA1819* were higher in geriatric elephants while *Schwartzia*, *Acinetobacter*, *Lysinibacillus*, *Solibacillus*, and *Bacteroidetes_BD2-2* were lower in geriatric elephants when compared to subadult and adult elephants (Fig. 3).

### Blood parameters of subadult and adult elephant

**Figure 4:**
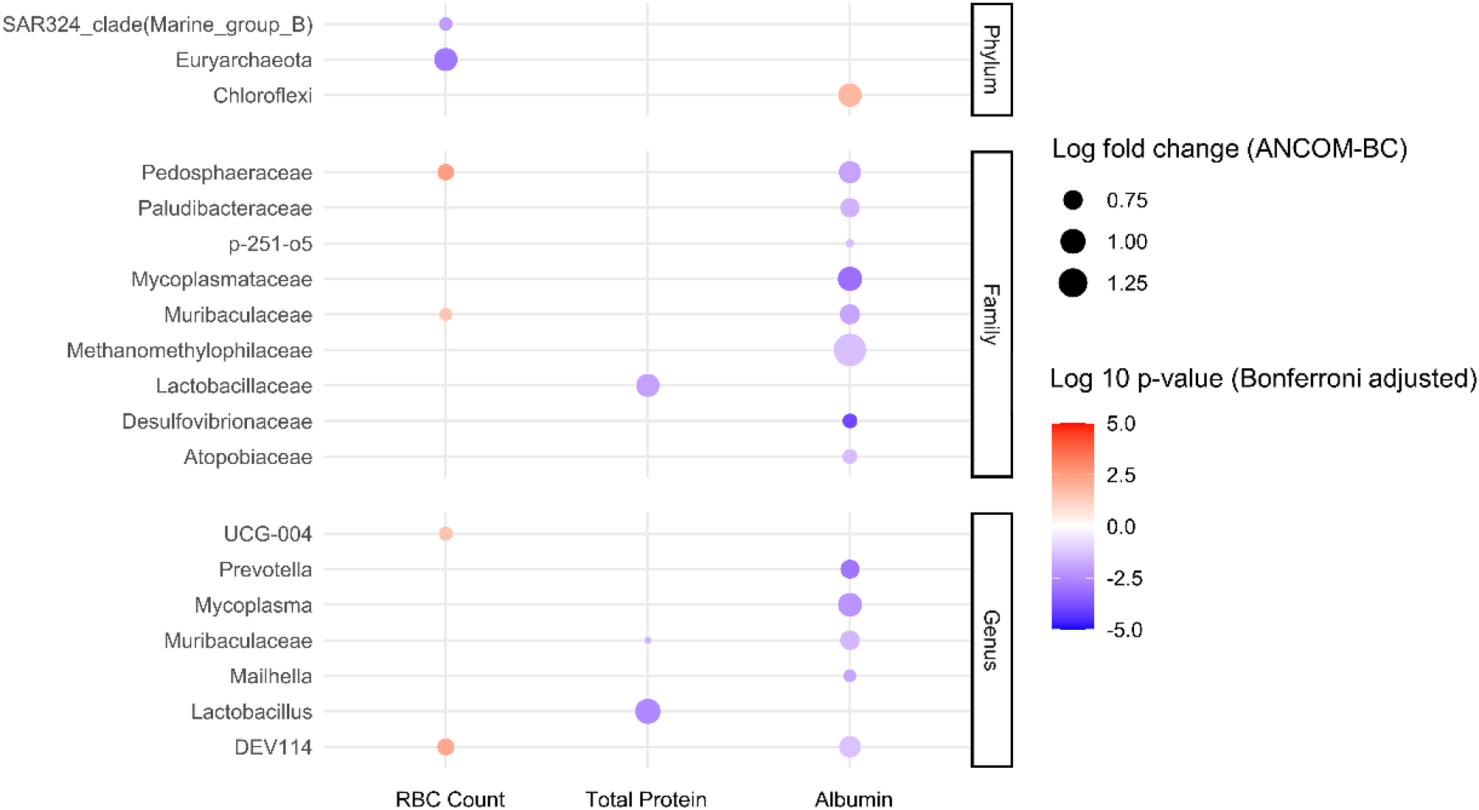
Log fold change of taxa in association with blood parameters in subadult and adult healthy captive elephants in Northern Thailand. Only log fold changes greater than 0.5 are shown in this figure. Full lists of significant associations are provided in Supplementary Table 4.

To further understand the association between gut microbiota and the health of elephants, the associations between gut microbiota composition and blood parameters were determined. All blood parameters and normal blood values are presented in Table 2. The hematological and biochemical parameters of subadult and adult elephants were within the normal range when compared to earlier research conducted by Mikota^24^. The serum lipid profiles including triglyceride (TG), total cholesterol (TC), high density lipoprotein (HDL), and low-density lipoprotein (LDL) were all determined to be within the normal range, when compared to the previous study by Norkaew, et al.^25^. In these analyses, the blood parameters were used as the numerical outcome in the ANCOM-BC analysis, and the results with a log-fold change of gut microbiota greater than 0.5 are shown in Fig. 4. The results showed that several gut microbiota of subadult and adult elephants showed a correlation with blood parameters including RBC count, total protein, and serum albumin. However, the correlation between gut microbiota and PC1 of overall blood parameters did not show the same significant taxa (Supplementary Fig. 1) which might indicate that the gut microbiota tended to correlate with specific blood parameters rather than the change of overall blood profile.

**Table 2:** Characteristics of blood parameters in subadult and adult elephants.

Parameters	Mean ± standard deviation	Mikota^24^	Norkaew et al.^25^
Pack cell volume (%)	35 ± 4	30–40	N/A
Hemoglobin (g/dl)	12.69 ± 1.58	11–15	N/A
RBC count (× 10^6^ cells/μl)	2.88 ± 0.4	2.5–5.0	N/A
MCV (fl)	123 ± 6	80–160	N/A
MCHC (g/dl)	35.9 ± 0.75	25–40	N/A
WBC count (cells/μl)	12,005 ± 2372	10,000–18,000	N/A
Segmented neutrophil (cells/μl)	2690 ± 898	2000–4000	N/A
Lymphocyte (cells/μl)	6193 ± 1805	5000–8000	N/A
Monocyte (cells/μl)	2725 ± 1333	2000–4000	N/A
Eosinophil (cells/μl)	339 ± 218	100–1000	N/A
Basophil (cells/μl)	202 ± 95	0–30	N/A
Platelet count (× 10^3^ cells/μl)	353 ± 72	100–600	N/A
BUN (mg/dl)	10 ± 3	5–20	N/A
Creatinine (mg/dl)	1.47 ± 0.28	1.0–2.0	N/A
AST (U/L)	17 ± 6	15–35	N/A
ALT (U/L)	2 ± 1	1.5–3.0	N/A
ALP (U/L)	97 ± 49	60–450	N/A
Total protein (g/dl)	8.55 ± 0.63	6–12	N/A
Albumin (g/dl)	3.24 ± 0.35	1.5–3.5	N/A
CK(U/L)	166 ± 69	50.0–250.0	N/A
TC (mg/dl)	45 ± 10	N/A	24.4–48.5
TG (mg/dl)	23 ± 14	N/A	18.1–43.0
HDL (mg/dl)	12 ± 2	N/A	9.2–19.0
LDL (mg/dl)	29 ± 8	N/A	14.3–41.1

*RBC* red blood cell, *MCV* mean corpuscular volume, *MCHC* mean corpuscular hemoglobin concentration, *WBC* white blood cell, *BUN* blood urea nitrogen, *AST* aspartate transaminase, *ALT* alanine transaminase, *ALP* alkaline phosphatase, *CK* creatine kinase, *TC* total cholesterol, *TG* triglyceride, *HDL* high density lipoprotein, *LDL* low density lipoprotein.

The number of RBC showed a positive association with families *Pedosphaeraceae* and *Muribaculaceae*, and genera *DEV114* and *UCG-004* (Fig. 4). However, the phyla SAR324_clade(Marine_group_B) and Euryarchaeota showed a negative association with RBC count (Fig. 4). The total protein content showed a negative association with the genus *Lactobacillus* and the family *Lactobacillaceae* (Fig. 4)*.* The genera *Mycoplasma* with corresponding family *Mycoplasmataceae*, *Mailhella* with corresponding family *Desulfovibrionaceae*, *DEV114* with corresponding family *Pedosphaeraceae*, *Muribaculaceae* with corresponding family *Muribaculaceae*, and *Prevotella* were negatively associated with the level of serum albumin (Fig. 4). The family *p-251-o5*, *Atopobiaceae*, *Paludibacteraceae*, and *Methanomethylophilaceae* also showed a negative association with plasma albumin level (Fig. 4). However, at the phylum level, Chloroflexi showed a positive association with serum albumin (Fig. 4). The other blood parameters are listed in Table 2. The relationships of less significant association with the composition of gut microbiota and the details are shown in Supplementary Table 4. Furthermore, several blood parameters, such as red blood cell (RBC) count, mean corpuscular volume (MCV), white blood cell (WBC) count, and creatinine, exhibited a significant distinction (p < 0.05) between females and males (see Supplementary Table 5).

## Discussion

This study is the first study to characterize the gut microbial community in captive Asian elephants of different ages. In addition, the association between the gut microbiota and blood parameters was analyzed. The major findings of this study include: (1) the diversity and the composition of gut microbiota were different between age groups and significant differences were observed between the infant calves and suckling calves, and between adult and geriatric elephants; (2) the composition of the gut microbiota of subadult and adult elephants was high abundance of Firmicutes, followed by Bacteroidetes, and Actinobacteria; and (3) several gut microbiota in adult elephants were associated with blood parameters such as RBC count, total protein, and albumin. Our findings suggest that gut microbial composition in elephants might be dependent not only on the types of food consumed, but also on the quality of the food, as has been observed in other animals.

Infancy is a critical time for the gut microbiome to grow since it shapes the host immune system and stabilizes the metabolic condition^26,27^. Infant calves consume milk as their main source of energy, and the beta diversity analysis showed that the samples in infant calves were separately clustered from the rest of the older elephant groups in PCoA plots and the ANCOM-BC analysis also revealed that the composition of the gut microbiota composition was distinct. Milk fermenting bacteria including genus *Bifidobacterium* of family *Bifidobacteriaceae* together with genus *Akkermansia* were significantly higher as regards abundance in infant calves. This finding suggested that maternal milk was the key component determining the microbial composition of the gut in the infants enabling the newborn's intestines to adapt to the environment. Oligosaccharides and milk glycans are some of the major solid bioactive components in maternal milk; however, newborns were unable to digest and absorb them. When they passed through the gut lumen, these compounds have been served as prebiotics by shaping and stabilizing the microbiota in early life^28^. In the milk of Asian elephants, 40% of milk carbohydrate content was found to be oligosaccharides, a higher concentration than in other animal milk^29^. Milk oligosaccharides were mainly utilized by glycan-degrading enzymes, encoded by the genome of an infant-associated *Bifidobacterium* as well as other bacterial species within *Bacteroides* spp. and *Akkermansia* spp.^30,31^. It is worth noting that the milk utilizing bacteria such as *Bifidobacteriaceae* were still higher in suckling and weaned calves when compared to adult elephants. However, during the pre-weaning period, the composition of the gut microbiota is very dynamic and unstable^32,33^. As elephant calves are completely weaned at age 3 years old, the abundance of milk utilizer bacteria declined relative to earlier infantile and suckling stages. This finding indicated that the milk from the mother elephants had a significant influence on microbial composition throughout early life.

Changes in the fecal microbial composition after the infantile life of elephants were observed in suckling calves or at the age of around four months due to the beginning of plant-based diet consumption. These elephants gradually switched from consuming milk to a variety of high-fiber diets, until completely weaned. Consequently, the gut microbiota of suckling and weaned calves were approaching the adult-like pattern as demonstrated by the overlapping of the composition of the gut microbiota of sucking and weaned calves with adult elephants as shown in the beta diversity analysis. As expected, the fiber-fermenting bacterial families including *Spirochaetaceae, p-251-o5, Paludibacteraceae, Planococcaceae,* and *Gastranaerophilales,* which were significantly low in abundance in the infant elephants, showed no significant difference in suckling and weaned calves when compared to adult elephants. Furthermore, there was a higher abundance of SCFA-producing bacteria including genera *Lachnospiraceae_NK3A20_group* in both suckling and weaned calves, and [*Ruminococcus]_gauvreauii_group* and *Syntrophococcus* in weaned calves. One study described evidence of a correlation between the genera *Lachnospiraceae_NK3A20_*and [*Ruminococcus]_gauvreauii_groups* with the concentration of volatile fatty acids and levels of microbial crude protein in the rumen of pre-weaning lambs^34^. In addition to the introduction of forage feeding in herbivores, coprophagia events had an impact on the foal microbiome which promoted the establishment of suitable gut microbial flora in young animals optimizing gut health during the pre-weaning period^9,35^. Coprophagic behavior was normally observed in juvenile elephants in the first 4–6 months of life^36^. Conceivably, the introduction of a high-fiber diet and coprophagia might shape the composition of the gut microbiota instigating an adult-like profile even though young elephants were still suckling.

In this study, the adult elephant’s main roughage consisted of Napier grass and corn stalks. As expected, a significant amount of fibrolytic bacteria were observed in adult elephants. The presence of *Lachnospiraceae* was necessary for the breakdown of complex carbohydrates in grass^37,38^. In addition, it has been shown that *Lachnospiraceae* are the main producers of SCFAs, especially butyrate which serves as a source of nutrients and growth factors for healthy gut epithelia and also contributes to the prevention of inflammation^39^. The family *Oscillospiraceae* together with the genera *NK4A214_group* were likely to be able to utilize intestinal host glycans and produce the important SCFAs such as butyrate and their metabolites^40^. The genus *Sarcina* of the family *Clostridiaceae*, a cellulase-producing bacteria with carbohydrate fermentative metabolism, was also dominant in adult elephant feces. *Christensenellaceae R-7_ group*, a genus in the family *Christrensenellacease* which presented as a prominent bacterium genus in adult elephant feces, was reported to be associated with the host’s body mass index (BMI) and leanness in humans^41^.

The geriatric elephants showed a distinct gut microbiota profile as demonstrated by decreasing alpha diversity and the separated cluster of the microbes in the geriatric elephants from the other age groups in beta diversity analysis. The differential abundance analysis also revealed a higher abundance of the family *Lactobacillaceae* and the genus *Lactobacillus* in geriatric elephants when compared to adult elephants. *Lactobacillus* has been identified as one of the major bacterial taxa in the healthy horse^42^. Interestingly, overconsumption of high-starch diets and oligofructose has been associated with the overgrowth of *Lactobacillus* and has been observed in horses with colic frequently leading to the development of laminitis^42,43^. Consumption of elephant concentrate pellets by most geriatric elephants, which contained crude protein 11.6% and nitrogen-free extract 46.4% of dry matter^44^, might be associated with the higher abundance of *Lactobacillus* than in the subadult and adult group. Furthermore, the archaea *Methanobrevibacter spp.* of phylum Euryarchaeota, after having dipped in the middle age group, shifted to an increase again in geriatric elephants. This was in accordance with elderly macaques, which indicated a positive association of these methanogens with host aging^45^, and a high abundance of centenarian human gut microbiota^46^. The methanogens community has been linked to human health and various diseases. Decreased levels of methanogens have been observed in conditions like Crohn's disease, ulcerative colitis, and malnutrition, while increased levels have been noted in cases of constipation, diverticulosis, inflammatory bowel disease, and irritable bowel syndrome^47,48^. The decline in gut microbiota diversity in geriatric elephants might be due to the loss of the last molar teeth (M6) at the age of 50 leading to poor mechanical and hence chemical digestion^49^ and resulting in GI problems such as: colic, impactions, malabsorption and weight loss. According to study by Millette, et al.^50^, dental impairment led to larger food particles in ring-tailed lemur’s feces, potentially resulting in less efficient food utilization. Additionally, Xu, et al.^51^ found that increased tooth loss led to dietary changes due to reduced chewing abilities, which associated with lower diversity of gut bacteria. This might indicate lifestyle-related chronic inflammation in the colon, potentially influenced by both anti-inflammatory and possibly pro-inflammatory gut microbiota. Therefore, the diminished chewing abilities resulting from the loss of last molar teeth not only elevate the risk of obstructive colic issues in Asian elephants, but also lead to a decrease in gut microbiota diversity. However, further research should discuss and gather more evidence on how a poorly digested diet affects the gut microbiota.

In this study, the evaluation of the subadult and adult elephants' health status was primarily based on the assessment of body condition scores, as described by Morfeld, et al.^52^ which showed the body condition score of 3–4 was considered to be normal and healthy according to previous study by Bansiddhi, et al.^53^. Moreover, blood parameters of elephants in the subadult and adult groups were in the normal range of the hematological reference interval, as indicated in the Table 2. No illnesses had been reported 6 months before the investigation. In the mature period of life, several gut bacteria were found to show significant correlations with RBC count, total protein, and albumin. The gut microbiota was recognized as a virtual metabolic organ with a pivotal role in regulating immune function and hematopoiesis through molecular mechanisms. Microorganisms and their by-products entered the circulation where they were detected by Toll-like receptors (TLRs), initiating signaling pathways leading to the production of inflammatory cytokines in response to pathogens or related metabolites^54^. However, information regarding these correlations was scarce in elephant research and could be found only in previous studies of other species, such as mice, pigs, and humans. Our investigation revealed that genera *Mycoplasma* and *Prevotella* had negative associations with the level of serum albumin. According to a previous study, *Mycoplasma* presumably plays an important role in the etiology and pathology of primary biliary cirrhosis in humans^55^. *Prevotella* is believed to benefit host health as it can produce a significant amount of SCFA in pig model^56^, and sufficient to have anti-inflammatory effects in mouse model^57^. Nonetheless, *Prevotella* was reported to be associated with chronic inflammatory conditions and was found to have an increase in abundance in patients with chronic liver disease with advanced fibrosis^58,59^. We also found the bacterial family *Lactobacillaceae,* in particular genus *Lactobacillus,* was negatively associated with total protein in subadult and adult elephants. *Lactobacillus* is recognized as a beneficial microbe in humans and animals due to its involvement in immunity, metabolism, and maintaining the gut microbiota ecosystem^60^. This study determined the correlations between gut microbiota, RBC count, total protein, and serum albumin. Nevertheless, it remains uncertain whether alterations in the blood parameters were directly influenced by shifts in the gut microbiome or were a consequence of pathological changes. Future mechanistic studies into associations between bacterial taxa and blood parameters are warranted to delineate their significance and implications in elephant health issues.

The composition of gut microbiota in elephant can be influenced by the consumption of drinking water. Drinking water from different source were associated with differences in gut microbiota composition and be an important factor in shaping the gut microbiome^61^. Moreover, there was the chance of transfer of microorganisms from the drinking water source from one animal to the others^62^. Therefore, when elephants share a common water source, they may inadvertently ingest microbes present in the water. This can lead to the introduction of similar microbial species into their digestive systems. Future research would be needed to comprehensively understand the extent of this influence.

This study demonstrated the composition of gut microbiota in captive Asian elephants by using the 16s rRNA data from a number of elephants which could provide important information on the composition and diversity of these gut microbiota. While it is regrettable that the study did not involve a direct analysis of the food to determine the exact nutrient content ingested by the elephants, we attempted to estimate nutritional values based on the known dietary preferences of elephants. The future study to extrapolate nutrient composition from food in the elephants should be investigated. Notably, accounting for seasonal shifts in elephant diets, which may impact gut microbiota variations, is essential. This comprehensive approach significantly augments the precision of microbiota-related findings. Furthermore, while our sample encompassed a substantial number of subadult and adult elephants, the representation of other age groups, particularly infants, was limited. Extending the range of data acquisition or collaborating with other elephant camps might increase the number of young elephants. The definition of age group in this study was mainly based on age range, however other factors might be more suitable when categorizing the groups based on the biology of elephants and should be included in further study. In addition, this study exclusively focused on healthy elephants across all age groups; future investigations incorporating both healthy and diseased elephants may afford greater insights into the multifaceted roles of gut microbiota in the health and biology of elephants.

## Conclusion

Our findings indicate that the diversity and relative abundance of gut microbiota in captive Asian elephants were affected by age-related changes. This knowledge will inform more appropriate food management to facilitate the development of more beneficial bacteria for each elephant at the different stages of their life. The elephants in this study, all lived in similar environments and were raised and handled similarly. In the future, a comparison of zoo husbandry conditions and wild environments, as well as differences in food management and actual nutrient composition, should be further investigated to promote the most effective management and nutrition for a healthy gut in captive elephants.

## Methods

This study was approved by the Institutional Animal Care and Use Committee, Faculty of Veterinary Medicine, Chiang Mai University, Chiang Mai, Thailand (FVM-ACUC; R3/2563) and all experiments were performed in accordance with relevant guidelines and regulations. All methods used in this study are reported in accordance with ARRIVE guidelines. Fecal samples of approximately 50 g, were one time collected from 134 healthy captive elephants aged between two months and 67 years old working in tourist camps in Chiang Mai and Lampang provinces, during January–December 2020. Only one sample was collected per individual. All elephants (n = 134) in this study were categorized into five groups based on nutrition and growth pattern, including one infant calves (1 day to 4 months old, n = 6); two suckling calves (4 months to 3 years old, n = 12); three weaned calves (3–10 years old, n = 27); four subadult and adult elephants (10–55 years old, n = 78); and five geriatric elephants (more than 55 years old, n = 11). The group sizes in each category were determined based on the existing elephant population in the Northern region. Furthermore, a significant number of samples of subadult and adult elephant groups were collected to ensure representation across a broad range of ages. Individual body condition score was estimated as scored by Morfeld, et al.^52^ which permit a visual evaluation of the backbone, rib bone and pelvic bone areas, and ranges from 1 to 5 (where 1 signifies the thinnest and 5 signifies the fattest), with an optimal score of 3 (Table 3). The information regarding food and water consumption in each group is presented in Table 3.

**Table 3:** General characteristics and details of food and water consumption of healthy captive elephants in Northern Thailand categorized by age group.

	G1 (infant calves)	G2 (suckling calves)	G3 (weaned calves)	G4 (subadult and adult elephant)	G5 (geriatric elephants)
General information					
N	6	12	27	78	11
Age (years)	0.20 ± 0.00	1.58 ± 0.50	5.70 ± 1.80	31.53 ± 11.70	58.90 ± 3.70
Male:female	4:2	6:6	12:15	14:64	0:11
Body condition score	3.00 ± 0.00	3.08 ± 0.30	3.33 ± 0.60	3.62 ± 0.70	3.18 ± 0.40
Diet					
Breast milk frequency (time/day)	8	8	0	0	0
Roughage (kg/day)	–	85.00 ± 25.05	157.96 ± 32.56	163.91 ± 32.87	180.00 ± 21.21
Roughage frequency (time/day)	–	3.67 ± 0.49	3.85 ± 0.82	4.12 ± 0.77	4.36 ± 0.50
Pellets (kg/day)	0	0.5	1	0	1
Pellets frequency (time/day)	0	1	1	0	1
Supplements (kg/week)	0	35.00 ± 30.90	26.67 ± 33.28	78.46 ± 62.15	63.64 ± 60.54
Supplements frequency (time/week)	0	3.50 ± 3.09	1.5 ± 1.69	4.25 ± 3.01	3.18 ± 3.027
Source of water consumption					
River (%)	0	41.18	30.64	24.07	31.82
Pond (%)	0	23.53	25.81	27.78	18.18
Mountain water supply (%)	100	35.30	38.71	38.89	31.82
Tap water (%)	0	0	4.84	9.26	18.18
Frequency (time/day)	3	3	3.11 ± 0.32	3.19 ± 0.40	3.36 ± 0.50

For the first four months of their lives, maternal milk was the major source of nutrition for infant calves. Suckling calves were still consuming breast milk but also began to consume fruit supplement as solid food. Weaned calves and subadult and adult elephants were fed with napier grass (*Pennisetum purpureum*) and corn stalks (*Zea Mays* L.) as their main roughage. Geriatric elephants were fed with roughage that had been chopped into small pieces and mixed with pellets. Elephant concentrate pellets (Erawan^®^, CPF, Thailand) were given as a daily supplement to the geriatric elephants and some suckling and weaned calves. Seasonal supplements including banana, sugar cane, mango, tamarind, and watermelon were provided as supplements to all elephants except newborn calves. Elephants could access the water sources ad libitum or as given by the mahout, which was 3–4 times a day. Water was sourced from mountain streams, rivers, or ponds (Table 3).

All the elephants had been housed and had worked in the camp for over 6 months, with elephant calves born and raised in the original camp without relocation. Calves and juveniles were housed together with their mother without interacting with the tourists. The adult elephants routinely worked with tourists, trekking, participating in elephant shows, or for observation between 8:00 and 15:00 for no more than 5 h per day^63^. The geriatric elephants were not involved in any physical activities with tourists. The information on types of work, habitat use, food intake, and foraging behavior was collected individually.

The elephants were verified as being clinically healthy and evaluation of body condition by experienced elephant veterinarians, based on history and clinical examination, with no reports of GI issues or antibiotic or drug administration for at least six months before the beginning of the study.

### Blood collection

A 10 mL blood sample was collected from the ear vein of each elephant in the subadult and adult group, since there was reluctance among certain elephant owners to permit blood sampling from young elephants. These young elephants were not well trained for blood collection, and owners were concern that it might induce stress in the animals. Therefore, we had chosen to focus on adult and subadult elephants for the haematological and biochemical parameters in order to investigate the correlation between gut microbiota and elephant health. In addition, blood and fecal samples were collected concurrently to ensure that the blood profile accurately reflects the relationship with gut microbiota. Blood samples were submitted to the Veterinary Diagnostic Laboratory of Faculty of Veterinary Medicine, Chiang Mai University, Thailand within 24 h of collection. Blood samples were analyzed using the Auto Haematology Analyzer (Mindray BC5300, Mindray Medical, Thailand) and using the Biochemical Analyzer Vitalab Flexor XL (Vital Scientific NV, Netherlands). Hematology parameters i.e. packed cell volume (PCV), hemoglobin, RBC count, mean corpuscular volume (MCV), mean corpuscular hemoglobin concentration (MCHC), White Blood Cell count (WBC count), and platelet count, and biochemical parameters i.e. Blood Urea Nitrogen (BUN), creatinine, aspartate aminotransferase (AST), alanine aminotransferase (ALT), and alkaline phosphatase (ALP), creatine kinase (CK), total protein and albumin were analyzed.

We also measure blood lipid concentrations to evaluate the overall health status of elephants, especially in instances of elevated body condition or obesity. The serum lipid profiles were assessed were analyzed as described by Norkaew, et al.^25^. Serum lipids were quantified using an Automated Clinical Chemistry Analyzer (Sysmex; BX-3010, Sysmex Corporation of Japan)^21^. TC levels were determined through a cholesterol oxidase–peroxidase (CHOD-PAP) method. TG were assessed using a colorimetric enzymatic test involving glycerol-3-phosphate-oxidase (GPO) method. LDL and HDL levels were measured using a homogeneous method, with a sensitivity of 0.1 mmol/L (99.7% confidence). The lowest detectable concentration for TC was 0.1 mmol/L (99.7% confidence), and 0.05 mmol/L for LDL and HDL.

### Fecal samples collection and analysis

Fresh fecal samples (approximately 50 g) were collected directly from the rectum or immediately after defecation and stored at −20 °C within 3 h of collection until DNA extraction. After the samples were thawed, indigestible roughage such as grass, leaves, fruit seeds, and peel were separated from the stool contents. Up to 250 mg of prepared stool was used for DNA extraction. Bacterial genomic DNA was extracted from elephant fecal pellets using a commercial genomic DNA isolation kit [QIAamp PowerFecal Pro DNA Kit (Qiagen), Germany]. The extracted bacterial genomic DNA were exposed to an amplification process of hypervariable region V3–V4 of 16s rRNA and then underwent NGS methods. DNA amplification, data quality control methods, and sequencing were conducted by Novogene Inc (Singapore City, Singapore). A Double-blind study with samples categorization was used to avoid bias.

### Sequencing data analysis

The extracted bacterial genomic DNA were exposed to an amplification process of hypervariable region V3–V4 of 16s rRNA (F 5′-CCTAYGGGRBGCASCAG-3′, R 5′-GGACTACNNGGGTATCTAAT-3′) and then underwent NGS methods. DNA sequence data from NGS were processed using the Quantitative Insights Into Microbial Ecology 2 (QIIME2-2021.4) open-source software^64^. The raw datasets containing pair-ended reads and quality scores were merged, denoised, trimmed according to quality scores, and assigned to amplicon sequence variants (ASV) by using q2‐dada2 plugin^65^. A feature table including the number of each ASV per sample was also generated^66^. The ASVs were aligned with mafft and used to generate a phylogenetic tree for further analysis^67,68^. The diversity analyses were conducted with rarefication at the sequencing depth of 56,700 as this is the maximum number to retain all samples in this study^69^. The alpha diversity measures, including observed species, Pielou’s evenness^70^, Faith’s phylogenetic diversity^71^, and Shannon’s index^72^ were calculated and analyzed using Kruskal–Wallis test in QIIME2. The beta diversity including Bray Curtis, Jaccard, unweighted UniFrac^73^, and weighted UniFrac^74^ distance matrices were analyzed and were illustrated as principal coordinate analysis (PCoA). The taxonomy was assigned to ASVs by using q2‐feature‐classifier classify‐sklearn naïve Bayes taxonomy classifier^75^ by using the data reference from SILVA database version 138^76^. For visualization of population structure and relative abundance, taxonomical bar plots indicating relative abundance at phylum, class, and genus levels of each sample from the different age groups were generated. The differences in taxa abundance between categories and the association between taxa and blood parameters were estimated with a statistical framework: analysis of composition of microbiomes with Bias Correction (ANCOM-BC)^77^. The data visualization was conducted through R version 4.1.1 by using package qiime2R.

### Supplementary Information

Supplementary Table 1.

Supplementary Table 2.

Supplementary Table 3.

Supplementary Table 4.

Supplementary Table 5.

Supplementary Figure 1.

## Data Availability

The datasets generated and/or analyzed in this study are available in the NCBI sequence read archive under the Accession Number PRJNA1005601.
