# Supplementary material for: Characteristics of gut microbiota in captive Asian elephants (Elephas maximus) from infant to elderly
Source: Sci Rep. 2023 Dec 27;13:23027. doi: 10.1038/s41598-023-50429-1 (PMC10754835; doi:10.1038/s41598-023-50429-1)
Supplement: Supplementary file 1 — Supplementary Table 1. [file 41598_2023_50429_MOESM1_ESM.docx]

**Supplementary Table 1.** Permutational Analyses of Multivariate Dispersions (PERMDISP) of beta diversity between age group of elephants

|  | **F-value** | **p-value** |
| --- | --- | --- |
| **Bray Curtis** | 2.351951 | 0.045 |
| **Jaccard** | 11.846888 | 0.001 |
| **unweighted UniFrac** | 13.829536 | 0.001 |
| **weighted UniFrac** | 2.642909 | 0.032 |
