# Supplementary material for: Characteristics of gut microbiota in captive Asian elephants (Elephas maximus) from infant to elderly
Source: Sci Rep. 2023 Dec 27;13:23027. doi: 10.1038/s41598-023-50429-1 (PMC10754835; doi:10.1038/s41598-023-50429-1)
Supplement: Supplementary file 2 — Supplementary Table 2. [file 41598_2023_50429_MOESM2_ESM.docx]

**Supplementary Table 2.** Mantel test between beta diversity and age of subadult and adult elephants both in all sample and geriatric elephant excluded data.

|  | **All sample** | | **Geriatric elephant excluded** | |
| --- | --- | --- | --- | --- |
|  | **Spearman’s rho** | **p-value** | **Spearman’s rho** | **p-value** |
| **Bray Curtis** | 0.304374 | 0.001 | 0.239347 | 0.001 |
| **Jaccard** | 0.278888 | 0.001 | 0.188315 | 0.001 |
| **unweighted UniFrac** | 0.233651 | 0.001 | 0.124296 | 0.001 |
| **weighted UniFrac** | 0.219175 | 0.001 | 0.157485 | 0.001 |
