# Supplementary material for: Characteristics of gut microbiota in captive Asian elephants (Elephas maximus) from infant to elderly
Source: Sci Rep. 2023 Dec 27;13:23027. doi: 10.1038/s41598-023-50429-1 (PMC10754835; doi:10.1038/s41598-023-50429-1)
Supplement: Supplementary file 5 — Supplementary Table 5. [file 41598_2023_50429_MOESM5_ESM.docx]

**Supplementary Table 5** Student's T-test of haematological parameters of subadult and adult elephants by sex

| **Parameters** | **Female**  **(n=52)**  **(mean±sd)** | | | **Male**  **(n=11)**  **(mean±sd)** | | | **p-value** |
| --- | --- | --- | --- | --- | --- | --- | --- |
| Pack cell volume (%) | 35.12 | ± | 4.46 | 37.00 | ± | 4.00 | 0.092 |
| Hemoglobin (g/dl) | 14.31 | ± | 12.35 | 13.31 | ± | 1.53 | 0.287 |
| RBC count (×10^6^ cells/μl) | 2.84 | ± | 0.38 | 3.10 | ± | 0.45 | 0.046* |
| MCV (fl) | 123.67 | ± | 5.42 | 120.05 | ± | 5.83 | 0.040* |
| MCHC (g/dl) | 35.87 | ± | 0.78 | 35.91 | ± | 0.63 | 0.430 |
| WBC count (cells/μl) | 12259.62 | ± | 2353.27 | 10903.64 | ± | 2130.35 | 0.039* |
| Segmented neutrophil (cells/μl) | 2775.19 | ± | 944.88 | 2460.00 | ± | 797.94 | 0.133 |
| Lymphocyte (cells/μl) | 6269.96 | ± | 1811.20 | 5752.91 | ± | 1719.45 | 0.192 |
| Monocyte (cells/μl) | 2804.40 | ± | 1301.29 | 2375.27 | ± | 1427.61 | 0.187 |
| Eosinophil (cells/μl) | 336.96 | ± | 215.56 | 327.50 | ± | 237.95 | 0.455 |
| Basophil (cells/μl) | 201.20 | ± | 94.65 | 203.00 | ± | 137.18 | 0.494 |
| Platelet count (×10^3^ cells/μl) | 356.19 | ± | 68.62 | 336.27 | ± | 84.28 | 0.238 |
| BUN (mg/dl) | 9.33 | ± | 2.82 | 10.76 | ± | 2.89 | 0.078 |
| Creatinine (mg/dl) | 1.44 | ± | 0.27 | 1.61 | ± | 0.22 | 0.019* |
| AST (U/L) | 16.85 | ± | 5.72 | 19.09 | ± | 8.53 | 0.210 |
| ALT (U/L) | 2.35 | ± | 1.10 | 2.45 | ± | 0.93 | 0.370 |
| ALP (U/L) | 97.50 | ± | 45.46 | 96.27 | ± | 64.87 | 0.477 |
| Total protein (g/dl) | 8.50 | ± | 0.62 | 8.73 | ± | 0.72 | 0.173 |
| Albumin (g/dl) | 3.91 | ± | 5.11 | 3.29 | ± | 0.36 | 0.196 |
| CK(U/L) | 169.62 | ± | 72.96 | 146.09 | ± | 42.42 | 0.081 |
| TC (mg/dl) | 44.31 | ± | 9.91 | 49.55 | ± | 9.79 | 0.065 |
| TG (mg/dl) | 23.56 | ± | 13.95 | 18.27 | ± | 13.70 | 0.133 |
| HDL (mg/dl) | 12.28 | ± | 2.52 | 11.69 | ± | 1.86 | 0.191 |
| LDL (mg/dl) | 28.17 | ± | 7.56 | 34.44 | ± | 8.36 | 0.019 |

* Indicates significant differences between sex-wise (p<0.05)

**Abbreviations**: RBC, Red blood cell; MCV, Mean corpuscular volume; MCHC, Mean Corpuscular Hemoglobin Concentration; WBC, White blood cell; BUN, Blood urea nitrogen; AST, Aspartate transaminase; ALT, Alanine transaminase; ALP, Alkaline phosphatase; CK, Creatine kinase; TC, Total cholesterol; TG, Triglyceride; HDL, High density lipoprotein; LDL, Low density lipoprotein.
