# Supplementary material for: Characteristics of gut microbiota in captive Asian elephants (Elephas maximus) from infant to elderly
Source: Sci Rep. 2023 Dec 27;13:23027. doi: 10.1038/s41598-023-50429-1 (PMC10754835; doi:10.1038/s41598-023-50429-1)
Supplement: Supplementary file 6 — Supplementary Figure 1. [file 41598_2023_50429_MOESM6_ESM.docx]

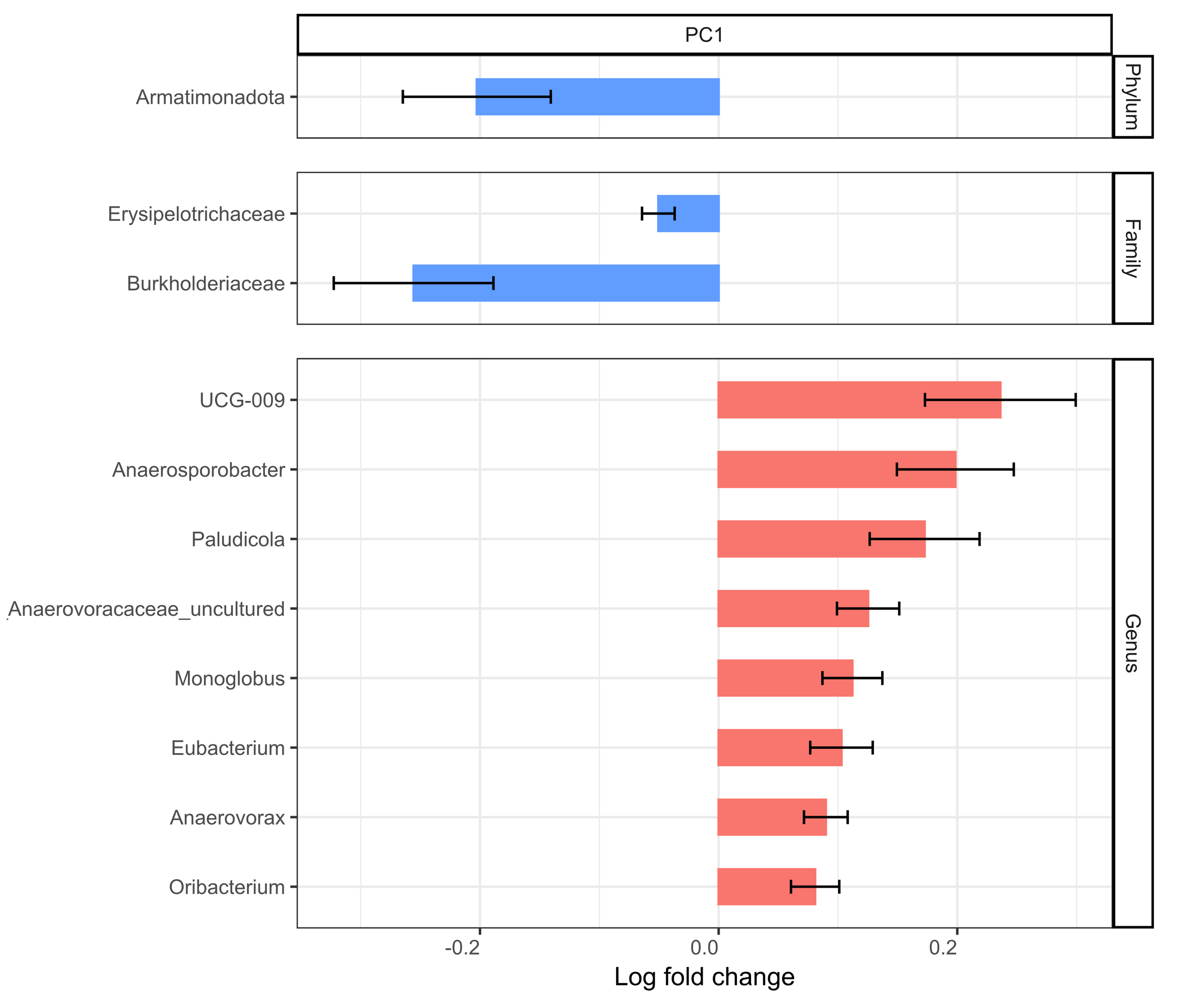


**Supplementay Figure 1.** Log fold change of taxa in association with principal component 1 (PC1) of blood parameters in subadult and adult healthy captive elephants in Northern Thailand. Hb, MCV, MCHC, WBC_count, segmented_neutrophil, Lymphocyte, Monocyte,Platelet, BUN, Cr, AST, ALT, ALP, TP, Albumin, CK, TC, TG, HDL, LDL were used to generate the PCA and PC1.
